# Digitally derived Ki-67 proliferation index for GastroEnteroPancreatic neuroendocrine neoplasms

**DOI:** 10.3389/pore.2025.1612248

**Published:** 2026-01-07

**Authors:** Tamás Micsik, Lilla Csellár, Árpád V. Patai, Anna Jakab, Viktor Jónás, Béla Molnár

**Affiliations:** 1 Department of Pathology and Experimental Cancer Research, Semmelweis University, Budapest, Hungary; 2 Interdisciplinary Gastroenterology Working Group, Semmelweis University, Budapest, Hungary; 3 Department of Surgery, Transplantation and Gastroenterology, Semmelweis University, Budapest, Hungary; 4 Department of Pathology, Forensic and Insurance Medicine, Semmelweis University, Budapest, Hungary; 5 3DHistech Ltd., Budapest, Hungary

**Keywords:** computational pathology, digital pathology, GEPNEN, Ki-67, machine learning

## Abstract

Ki-67 proliferation indices (PIs) define the grading of GastroEnteroPancreatic NeuroEndocrine Neoplasms (GEPNENs) and are crucial for therapeutic decisions. The precise Ki-67 assessment relies on manual counting, which is time-consuming, hardly accessible during routine pathological signout and thus usually replaced by the easier eye-estimation/balling method prone to interobserver variability and differences originating from the hot-spot size, localisation and tumor heterogeneity. These discrepancies can significantly affect the final PI resulting in misgrading of GEPNENs with potential adverse patient outcomes. In the era of digital pathology more and more applications are available to overcome this problem. In our retrospective study of 60 surgically resected GEPNEN cases, we tested the equivalence of traditional clinical (C) grading, manual counting with a MarkerCounter (MC) application and automatic grading with tumor recognition PatternQuant application with subsequent NuclearQuant (NQ) PI-assessment within 3DHistechs digital pathology platform. We found almost perfect agreement between the various grading methods (Spearman rank-order correlations: C vs. MC: *ρ* = 0.912, C vs. NQ: *ρ* = 0.883, MC vs NQ: *ρ* = 0.953) without clinically significant misgradings. Also the numerical values of the PIs derived with the various methods showed close correlations (Linear regression: C vs. MC: *r* = 0.952, C vs. NQ: *r* = 0.925, MC vs NQ: *r* = 0.978). The automated PI-assessment involved a mean 5-fold more tumor cells, better approximating the global/total Ki-67 PI, which was earlier shown to deliver more robust prognostic power and decreased interobserver variability. Furthermore, G3 tumors differed from G2 and G1 tumors in their cytomorphological parameterers: high grade tumors had significantly larger and more polymorphic, less regular tumor cell nuclei, which parameters could be also utilized for grading and/or prognostication purposes. Our study applied a simple, quick, easy-to-use, Machine Learning-based method that could be incorporated into routine digital pathology signout alleviating pathologists’ workload and increasing precision and recall rate.

## Introduction

GastroEnteroPancreatic NeuroEndocrine Neoplasms (GEPNENs) are rare and heterogeneous types of malignant tumors with variable behaviour [1]. GEPNENs are usually sporadic and well-differentiated, but inherited cases also exist. As GEPNENs can produce a variety of hormones, their symptoms are diverse. These tumors often remain undiscovered until late stages or are incidentally detected at earlier stages [2]. For survival estimation and prognostication TNM-staging and tumor grading are essential [3]. Proliferation is a reliable indicator of tumor growth capacity and serves as a valuable marker of the malignant potential. In grading GEP-NENs, Ki-67 proliferation index (PI) and mitotic counting are both accepted methods according to WHO guidelines, where the recommendation is to use the method indicating a higher grade [3, 4]. The WHO 2019 grading system is shown in Table 1. Ki-67-based grading proved to be more reliable than mitosis-based, most likely due to the high interobserver variability in counting the mitotic figures within a 2 mm^2^ area [5]. According to the WHO guidelines, Ki-67 PI should be defined on at least 500–2000 tumor cells in the most highly proliferating regions (hot-spots) of the tumor. However, the exact methodology for counting is not defined.

**TABLE 1:** Classification of GastroEnteroPancreatic NeuroEndocrine neoplasms according to WHO [4].

Classification of GastroEnteroPancreatic neuroendocrine Neoplasms (GEP-NENs) (2019 WHO)	Mitotic index (mitotic figures/10HPF)	Ki-67 proliferation index (%)
Well differentiated neuroendocrine tumors (WD-NET)		
Grade 1 (G1)	<2	<3
Grade 2 (G2)	2–20	3–20
Grade 3 (G3)	>20	>20
Poorly differentiated neuroendocrine carcinomas (PD-NEC)		
Grade 3 (G3)	>20	>20
Small cell neuroendocrine carcinoma (SC-NEC)		
Large cell neuroendocrine carcinoma (LC-NEC)		
Mixed neuroendocrine nonneuroendocrine neoplasias (MiNENs)		

The wide-spread “eye-balling” method, where a pathologist makes an estimate upon visual inspection, can produce ambiguous results; while “eye-counting”, when pathologists go cell by cell while counting, is a more precise but meticulous and time-consuming process. The most accurate approach is manual counting, which can be performed on printed images or digital slides. Despite its precision, manual counting is highly demanding, and rarely used in routine clinical practice [3, 6–8]. Furthermore, Ki-67 PI might also be affected by the size of hot spots and the selection of the region of interest (annotations used for counting) as the counting methods are not strictly defined [9–15]. The robustness of the prognostic power of Ki-67-based grading depends on multiple factors and often involves a laborious approach [10].

In the era of digital pathology and artificial intelligence the new technologies offer a wide range of applications designed to ease the pathologist’s workload. Whole slide imaging (WSI) enables precise manual counting of Ki-67 positive cells within rigorously defined hot spots using screens and digital platforms [16–21]. Automated image analysis can support tumor and hot-spot recognition and automate Ki-67 PI quantification, with a wide variety of analytical methods and software tools available to perform these tasks [22–27]. Moreover, artificial intelligence is now capable of predicting Ki-67 positive, proliferating cells directly from Hematoxilin-Eosin (HE) stained images [28, 29]. These new techniques are becoming more widely accessible and offer useful applications to take over these strenuous, yet clinically important and therapy-defining countings [1, 12, 13, 30–37].

In the current study we used a simple machine learning (ML)-based image analysis approach, for the automated tumor recognition and assessment of Ki-67 PI of GEPNEN slides. We compared the reliability of the automated method for tumor grading with digital manual counting and clinical counting methods. Furthermore, we also tested whether cytomorphological measurements could contribute to distinguishing between different grades of GEPNENs.

## Materials and methods

Ethical approval was received from the Hungarian Scientific Council National Ethics Committee for Scientific Research (no. 216/2020). The archive of the Department of Pathology and Experimental Cancer Research of Semmelweis University (Budapest, Hungary) was reviewed for GEPNENs diagnosed between 2009 and 2019. Biopsy samples were excluded to avoid insufficiently low cell numbers, resulting in a total of 60 surgical GEPNEN cases included in the study. The original diagnostical histology slides with HE and immunohistochemistry (IHC) stainings (Chromogranin A, Synaptophysin and Ki-67) were collected and scanned with Pannoramic 250 Flash II DX (3DHistech, Budapest, Hungary). For case handling and image analysis, 3DHistech’s digital platform, Slide Viewer was used.

**FIGURE 1:**
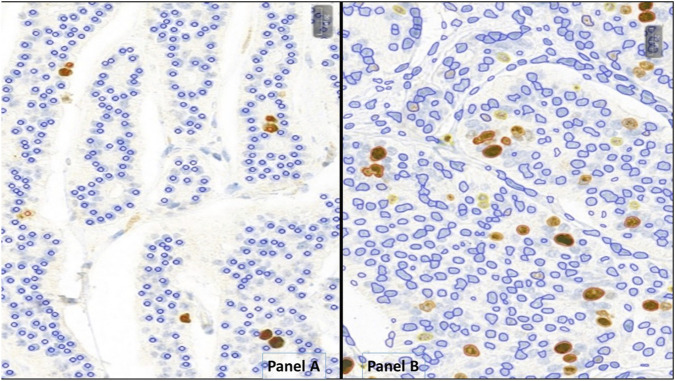
Neuroendocrine tumors analyzed with MarkerCounter **(A)** and NucelarQuant **(B)** applications of 3DHistech, Hungary. **(A)** red markers label the Ki-67-positive, proliferating tumor cells to count exact proliferation index. Blue markings are for the resting nuclei. Only tumor cells were marked, not the stroma or inflammatory ones. **(B)** the algorithm finds the tumor cell nuclei automatically and classifies those into positive (weak – yellow, medium – orange, strong - red) or negative (blue) classes and defines exact percentage of proliferation rate by counting the positive nuclei of the tumor cells.

On each slide, a 2 mm^2^ area within the tumors’ hot-spot region was manually annotated and used to conduct further analysis. Manual counting was performed using the MarkerCounter (MC) application (3DHistech, Budapest, Hungary) on the annotated hot-spot regions involving at least 2000 tumor cells by L. Cs under the supervision of T.M. With the MC-application, positive and negative markers were manually placed on each tumor cell nucleus (as shown in Figure 1A), and the program calculated the Ki-67 PI for each case based on these markings. Results from this approach are referred to as MC Ki-67/MC-Grading.

For automated Ki-67 quantification, PatternQuant and NuclearQuant applications were used on the 3DHistech platform. Both applications utilize machine learning-based algorithms and can be customized with adjustable parameters. PatterQuant is designed to identify and classify tissue elements based on colors and patterns. Small ROIs from tumorous and stromal regions were used to train the PatternQuant algorithm. These ROIs were iteratively added until the desired tumor segmentation fidelity was achieved. Subsequently, NuclearQuant application (developed to evaluate the nuclear staining on IHC-slides) was applied to automatically calculate PIs within the recognized tumor compartments. The algorithm recognizes cell nuclei and subsequently determines their IHC positivity and staining intensity, based on differences in color. In this case, the algorithm was trained on Ki-67-stained slides to differentiate between nuclei stained with DiAminoBenzidin (DAB) and background nuclei counterstained with Hematoxilin. Each nucleus is assigned as negative (blue marking) or weakly (yellow), moderately (orange) and strongly (red) positive and precise percentages are calculated for each category (Figure 1B). In addition, NuclearQuant performed cytomorphological measurements on each nucleus, such as cell perimeter, cell diameter, cell area and shape factor. The shape factor is a quantitative descriptor of nuclear roundness and regularity: a value close to 1 indicates a nearly perfect circle, while lower values reflect increasing irregularity of the nuclear shape. In our study PatternQuant was used firstly to identify tumor regions, followed by NuclearQuant for quantifying Ki-67 positive nuclei within the tumor regions and for performing cytomorphological measurements. After fine-tuning both applications, all settings were saved into a Scenario and subsequently all cases were analyzed with the same settings as a standardized approach. As in routine Ki-67 stainings any positivity in the nuclei should be evaluated as positive, the various positivity categories (low, moderate, strong) were combined. Results provided by this approach are referred to as NQ Ki-67/NQ-Grading.

Clinicopathological data, such as tumor localization, Ki-67 PI, and histological grade, were extracted from the original pathology reports. These data are referred to as Clinical Ki-67/Clinical Grading.

All data was stored in Microsoft Excel format for further statistical analyses performed with SPSS version 28.0.1.0 (IBM, Armonk, NY, United States). Differences between the various groups were calculated with Student t-probe. Correlations between the various proliferation indices were calculated according to Pearson, and non-continous variables, like grades, were compared with rank-order correlation methods: Spearman’s rho, Kendall-Tau, Cohen’s Kappa. Significance level was set to 5%, *p* < 0,05.

## Results

Of the 60 surgical cases, 7 were from the appendix, 2 from metastatic lymph nodes, 4 from the stomach, 13 from the pancreas, 12 from the large bowel, and 22 from small bowel. Based on the pathological reports, grades were as follows: 36 Clinical Grade 1, 9 Clinical Grade 2 and 15 Clinical Grade 3 GEPNENs. Table 2 shows the characteristics of the 60 cases.

**TABLE 2:** Ki-67 values and Grades ot the tumors from various origins with the different grading methods; SD: Standard deviation, G1,2,3: Grade 1, 2, 3.

GEPNEN localisation	Clinical Ki-67/Grading					MC Ki-67/Grading					NQ Ki-67/Grading				
	Average clinical Ki-67 PI (%)	SD	G1	G2	G3	Average MC Ki-67 PI (%)	SD	G1	G2	G3	Average NQ Ki-67 PI (%)	SD	G1	G2	G3
Appendix (n = 7)	1.21	0.39	7	0	0	0.59	0.70	7	0	0	0.51	0.52	7	0	0
Lymphnode metastasis (n = 2)	4.50	0.71	0	2	0	4.53	0.60	0	2	0	5.64	1.18	0	2	0
Stomach (n = 4)	34.00	30.28	1	1	2	25.22	35.82	1	2	1	27.18	35.33	1	1	2
Pancreas (n = 13)	19.05	29.12	7	3	3	10.52	25.71	8	4	1	9.52	21.39	10	2	1
Large bowel (n = 12)	41.78	28.77	2	0	10	53.89	27.55	2	0	10	61.05	32.77	2	0	10
Small bowel (n = 22)	1.91	1.14	19	3	0	1.55	2.19	20	2	0	1.14	2.00	20	2	0

**FIGURE 2:**
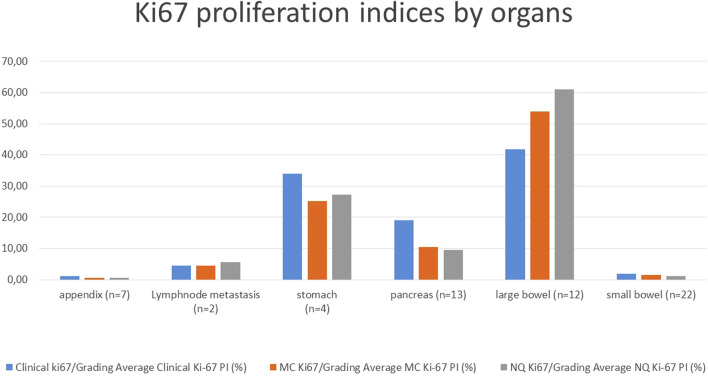
Average Ki-67 proliferation indices of the tumors from various origin with the different grading methods; MC: Marker Counter manual counting on digital slides, NQ: NuclearQuant automated counting, PI: Proliferation Index.

Average Ki-67 PIs and thus grades were calculated and grouped by organ with all methods: Clinical-Grading (as in the pathological report), MC-Grading (digital manual counting with MarkerCounter) and NQ-Grading (automated tumor-recognition with PatternQuant and subsequent NuclearQuant assessment). The Ki-67 PI results of the different methods are shown in Figure 2; Table 2, and compared in Table 3. Student’s t-probe showed significant difference between Clinical and NQ counting of Ki-67 PIs (1.21 vs. 0.51; *p* = 0.016), however, this difference was only numerical, around 1. All other proliferation indices were similar in average and the McNemar test did not show any significant differences between the various grading methods (Table 3).

**TABLE 3:** Differences between the Proliferation indices calculated with the various methods.

KI-67 PI %	t-probe, Clinical vs. MC	t-probe Clinical vs. NQ	t-probe, MC vs. NQ
Appendix	0.069	0.016	0.819
LN-metastasis	0.973	0.385	0.391
Stomach	0.741	0.795	0.940
Pancreas	0.460	0.380	0.915
Large bowel	0.345	0.169	0.569
Small bowel	0.505	0.129	0.522
All	0.725	0.565	0.817

McNemar extension	Clinical vs. MC	Clinical vs. NQ	MC vs. NQ
Appendix (n = 7)	1	1	1
Lymph node metastasis (n = 2)	1	1	1
Stomach (n = 4)	0.317	1	0.317
Pancreas (n = 13)	0.135	0.050	0.157
Large bowel (n = 12)	1	1	1
Small bowel (n = 22)	1	1	1
All samples (n = 60)	0.135	0.097	0.223

t-probe comparison of the Ki-67 proliferation indices and McNemar test comparing the various gradings; MC: Marker Counter, NQ: NuclearQuant assisted automated method. Significant difference is shown with bold letters, trends with italics.

We performed pairwise comparisons of the tumor grades provided by each method, as shown in the contingency Table 4. In case of appendix, lymph node metastasis, and large bowel tumors all grading methods delivered the same grade, achieving 100% agreement. Stomach and small bowel tumors also showed high concordance of the grading methods, 75%–100% and 91%–100% respectively. Out of 4 stomach cases that were investigated only a single case was misgraded by the automatic grading. Pancreatic tumors showed the lowest grading agreement (54%–85%) with 4, 6 or 2 out of the 13 cases misgraded, depending on the method. Overall, the 60 cases analyzed demonstrated a high level of grading concordance, 87%–95%, with 7, 8, and 3 cases misgraded out of the 60 cases. The highest match was found between MC and NQ-grading methods.

**TABLE 4:** Contingency table comparing the gradings performed upon the various methods of counting proliferation indices. The contingency tables show the pairwise comparative results of the various grading methods, with the matching grading percentages; C1-3: Clinical grades upon histology, MC1-3: Grades upon Marker Counter results, NQ1-3: Grades upon Nuclear Quant results. There are discrepancies, especially with pancreas cases, but altogether there is about 90% grading match across the various grading methods.

Appendix (n = 7)	100%	MC1	MC2	MC3	100%	NQ1	NQ2	NQ3	100%	NQ1	NQ2	NQ3
	C1	7	0	0	C1	7	0	0	MC1	7	0	0
	C2	0	0	0	C2	0	0	0	MC2	0	0	0
	C3	0	0	0	C3	0	0	0	MC3	0	0	0
LN-meta (n = 2)	100%	MC1	MC2	MC3	100%	NQ1	NQ2	NQ3	100%	NQ1	NQ2	NQ3
	C1	0	0	0	C1	0	0	0	MC1	0	0	0
	C2	0	2	0	C2	0	2	0	MC2	0	2	0
	C3	0	0	0	C3	0	0	0	MC3	0	0	0
Stomach (n = 4)	75%	MC1	MC2	MC3	100%	NQ1	NQ2	NQ3	75%	NQ1	NQ2	NQ3
	C1	1	0	0	C1	1	0	0	MC1	1	0	0
	C2	0	1	0	C2	0	1	0	MC2	0	1	1
	C3	0	1	1	C3	0	0	2	MC3	0	0	1
Pancreas (n = 13)	69%	MC1	MC2	MC3	54%	NQ1	NQ2	NQ3	85%	NQ1	NQ2	NQ3
	C1	6	0	0	C1	6	0	0	MC1	8	0	0
	C2	2	2	0	C2	4	0	0	MC2	2	2	0
	C3	0	2	1	C3	0	2	1	MC3	0	0	1
Large bowel (n = 12)	100%	MC1	MC2	MC3	100%	NQ1	NQ2	NQ3	100%	NQ1	NQ2	NQ3
	C1	2	0	0	C1	2	0	0	MC1	2	0	0
	C2	0	0	0	C2	0	0	0	MC2	0	0	0
	C3	0	0	10	C3	0	0	10	MC3	0	0	10
Small bowel (n = 22)	91%	MC1	MC2	MC3	91%	NQ1	NQ2	NQ3	100%	NQ1	NQ2	NQ3
	C1	19	1	0	C1	19	1	0	MC1	20	0	0
	C2	1	1	0	C2	1	1	0	MC2	0	2	0
	C3	0	0	0	C3	0	0	0	MC3	0	0	0
All samples (n = 60)	88%	MC1	MC2	MC3	87%	NQ1	NQ2	NQ3	95%	NQ1	NQ2	NQ3
	C1	35	1	0	C1	35	1	0	MC1	38	0	0
	C2	3	6	0	C2	5	4	0	MC2	2	7	1
	C3	0	3	12	C3	0	2	13	MC3	0	0	12

We compared the various grading methods using parametric (Cohen-kappa and Pearson) and, as grades are rather categorical than continuous variables, non-parametric, rank order correlations were also calculated (Kendall and Spearman coefficients) as shown in Table 5. Spearman’s correlation, the most widely used method yielded values of *ρ* = 0.912 between clinical and MC: *ρ* = 0.883 between clinical and NQ and *ρ* = 0.953 between MC and NQ-Grades. Other non-parametric correlation values ranged also from 0.848 to 0.963, well above 0.8, indicating almost perfect agreement, according to Landis et al. [38]. Cohen’s Kappa also showed substantial agreement between clinical and machine-derived methods (C vs. MC *Κ* = 0.786 and C vs. NQ *Κ* = 0.748), and almost perfect agreement between the two machine based methods (MC vs. NQ *Κ* = 0.978).

**TABLE 5:** Correlations between the different grading methods with rank-order calculations and between the variously calculated Ki-67 proliferation indices; MC: Marker Counter. NQ: NuclearQuant assisted automated method.

Grading Correlation	Clinical vs. MC	Clinical vs. NQ	MC vs. NQ
Pearson r	0.922	0.913	0.963
Kendall τ	0.881	0.848	0.940
Spearman ρ	0.912	0.883	0.953
Cohen’s K	0.786	0.748	0.903
Ki-67 PI values correlation	0.952	0.925	0.978

**FIGURE 3:**
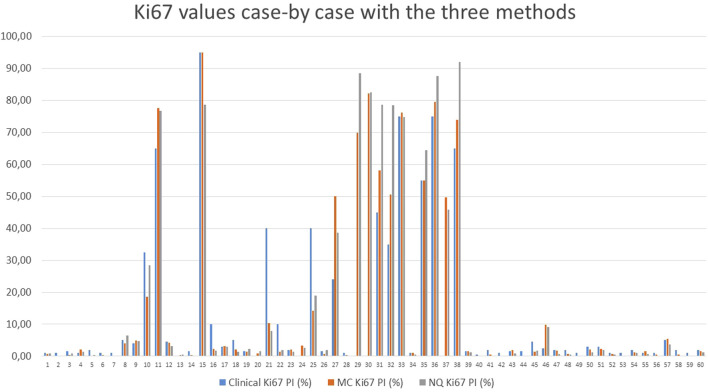
Comparison of the Ki-67 values of each case, counted with the different methods; MC: Marker Counter. NQ: NuclearQuant assisted automated method. PI: Proliferation Index.

The numerical Ki-67 PIs were compared using linear regression, revealing very strong correlations Clinical vs. MC *r* = 0.952; Clinical vs. NQ *r* = 0.925; MC vs. NQ *r* = 0.978, further supporting an almost perfect agreement between the different methods (Table 5; Figure 3). Neither McNemar’s test showed any significant differences between either of the grading methods, as shown in Table 3.

51 (85%) of the sixty cases showed complete match of gradings and only 9 (15%) cases revealed some discrepancy between the grading methods. These cases are shown on Table 6. Cases 17, 24, 50 were very close to the threshold between G1 and G2 tumors (Ki-67 PI 3%), where eye-balling might not be as precise as digital methods. Cases 16 and 22 were estimated to G2 tumors with Clinical Grading, though, digital measurement delivered lower proliferation rate putting the cases into G1 category with MC- and NQ-Grading. Case 10 was downgraded to G2 only with MC-grading, while Clinical and NQ-Grade remained G3. Cases 21 and 25 were clinically graded as G3, but both other methods put it into G2. Similarly Case 22 was downgraded from G2 to G1 by the digital methods. Case 46 is the most interesting, where clinical grade was G1, but both digital methods put the case into G2 group.

**TABLE 6:** Cases with discrepant gradings with either methods.

Case	GEPNEN localisation	Clinical grade	MC-grade	NQ-grade	Clinical Ki-67	MC Ki-67	NQ Ki-67
10	Stomach	3	2	3	32.50	18.59	28.39
16	Pancreas	2	1	1	10.00	2.18	1.73
17	*Pancreas*	*2*	*2*	*1*	*3.00*	*3.12*	*2.93*
21	Pancreas	3	2	2	40.00	10.40	7.94
22	Pancreas	2	1	1	10.00	1.38	1.98
24	*Pancreas*	*2*	*2*	*1*	*3.00*	*3.25*	*2.60*
25	Pancreas	3	2	2	40.00	14.18	19.02
46	Small bowel	1	2	2	2.50	9.79	9.19
50	*Small bowel*	*2*	*1*	*1*	*3.00*	*2.06*	*1.27*

Blue markings show the cases which resulted in lower grade with digital evaluation, whereas yellow marking show the only case which proved to be G2 with digital counting instead of the original Clinical G1 category. Red numbers show the cases close to the 3% thresholds, where the cause for discrepant grades was the lack of performing number rounding. MC: Marker Counter, NQ: NuclearQuant assisted automated method.

Independently of the grading methods (clinical, MC or NQ), Ki-67 PIs were significantly different between grades (Table 7).

**TABLE 7:** Comparison of the different cytomorphological parameters according to the different grading methods.

t-probe	Clinical Grading			MC-grading			NQ-grading		
Parameter	G1 vs. G2	G1 vs. G3	G2 vs. G3	G1 vs. G2	G1 vs. G3	G2 vs G3	G1 vs. G2	G1 vs. G3	G2 vs. G3
Clinical Ki-67 PI (%)	**0.009**	**p < 0.001**	**p < 0.001**	**0.049**	**p < 0.001**	**p < 0.001**	0.113	**p < 0.001**	**p < 0.001**
MC Ki-67 PI (%)	**0.005**	**p < 0.001**	**p < 0.001**	**0.002**	**p < 0.001**	**p < 0.001**	**0.004**	**p < 0.001**	**p < 0.001**
NQ Ki-67 PI (%)	**0.011**	**p < 0.001**	**p < 0.001**	**0.014**	**p < 0.001**	**p < 0.001**	**0.015**	**p < 0.001**	**p < 0.001**
TC nuclear-area, average	0.441	**p < 0.001**	**p < 0.001**	*0*.*077*	**p < 0.001**	**p < 0.001**	*0*.*091*	**p < 0.001**	**p < 0.001**
TC nuclear-area SD	*0*.*076*	**p < 0.001**	**p < 0.001**	**0.003**	**p < 0.001**	**p < 0.001**	**0.001**	**p < 0.001**	**p < 0.001**
TC nuclear perimeter, average	0.541	**p < 0.001**	**p < 0.001**	0.182	**p < 0.001**	**p < 0.001**	0.225	**p < 0.001**	**p < 0.001**
TC nuclear perimeter SD	*0*.*089*	**p < 0.001**	**p < 0.001**	**0.015**	**p < 0.001**	**p < 0.001**	**0.011**	**p < 0.001**	**p < 0.001**
TC nuclear shape-factor, average	0.926	**0.039**	**0.001**	0.840	**p < 0.001**	**p < 0.001**	0.820	**p < 0.001**	**p < 0.001**
TC nuclear shape-factor, SD	0.459	**0.011**	**p < 0.001**	0.936	**p < 0.001**	**p < 0.001**	0.578	**p < 0.001**	**p < 0.001**

​	Clinical G1	Clinical G2	Clinical G3	MC G1	MC G2	MC G3	NQ G1	NQ G2	NQ G3
TC nuclear-area, average, μm^2^	22.72	24.13	35.57	22.61	24.9	38.38	22.61	24.37	37.71
TC nuclear-area, SD, μm^2^	10.35	12.29	22.32	10.37	13.82	23.83	10.37	13.3	23.42
TC nuclear perimeter, average, μm	17.59	18.05	22.21	17.53	18.25	23.33	17.53	18.09	23.06
TC nuclear perimeter, SD, μm	4.65	5.15	8.04	4.67	5.42	8.55	4.67	5.29	8.4
TC nuclear shape-factor, average	0.89	0.89	0.84	0.89	0.89	0.83	0.89	0.89	0.83
TC nuclear shape-factor, SD	0.09	0.09	0.11	0.09	0.09	0.12	0.09	0.09	0.12

As we see, tumor cells showed significant differences in almost all cellular parameters in G1/G3 and also in G2/G3 relations, while G1 and G2 tumors showed less, still several significant differences. Italic values represent trends, while **bold** values show the significant differences. MC: Marker Counter. NQ: NuclearQuant assisted automated method. PI: Proliferation Index, TC: Tumor cell, SD: Standard deviation.

The average tumor cell count was 2029 (2000–2123) for MC-Grading and 10,629 (3691–22800) for NQ-grading.

**FIGURE 4:**
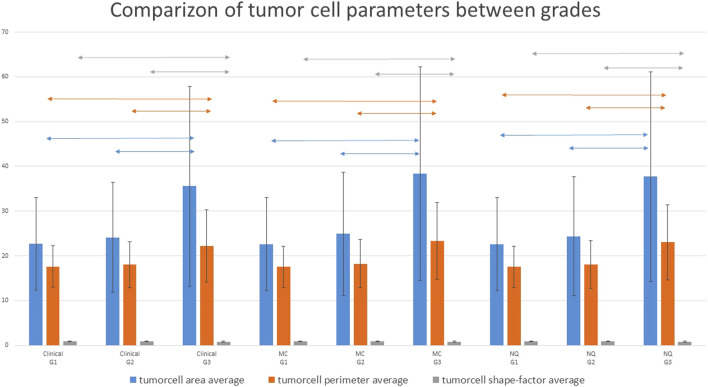
Comparizon of the various tumor cell nuclear parameters with the different grading methods. G1 and G2 cases showed significantly different parameters from G3 cases. whereas G1 and G2 cases did not differ significantly. Two-headed arrows show the significantly different groups (p < 0.05); MC: Marker Counter. NQ: NuclearQuant assisted automated method. PI: Proliferation Index.

Cytomorphological parameters within the tumors were compared in relation to clinical, MC and NQ-Grades. Tumor cell nuclear area, perimeter and shape factor significantly differed between G1 and G3 and also between G2 and G3 tumors with all grading methods. Higher grade tumor cells were usually bigger and less regular/circular. These differences were not significant in all parameters between G1 and G2 tumors. Table 7; Figure 4 shows how these parameters varied across the different grading groups.

## Discussion

Regarding Ki-67 PI, the appendiceal and small bowel tumors were mainly G1 GEPNENs. Pancreas and stomach tumors exhibited a wider range of PIs, however it should be noted that endoscopic biopsies of stomach GEPNENs were not included, thus the Type 1 gastric WDNETs with their typical low PIs were not present in our study. Large bowel tumors were overwhelmingly high grade tumors. Upper findings are similar to literature data [39].

The various Ki-67 PI counting methods delivered similar values and no significant differences were found except for appendix (Table 3), where NQ Ki-67 PI values were significantly lower than Clinical PI values and there was also a trend for having lower MC Ki-67 PIs than Clinical PIs. These slightly lower PIs were very close to or below 1%, where manual estimation is rather difficult and all appendiceal tumors remained in G1 category by either method, thus we claim that these variabilities around 1% were only numerical and had no clinical relevance.

Table 4 shows the contingency table with complete match regarding appendiceal (n = 7) and large bowel (n = 12) GEPNENs and lymph node metastases (n = 2). Stomach GEPNENs (n = 4) showed complete agreement between clinical and NQ-Grading, whereas a single case showed lower PI with MC than Clinical and NQ-Grading. Here MC-Grade was G2, while clinical and NQ-Grades were G3. This discrepancy can be explained by the hot-spot size and localisation on which MC-grading was performed.

Pancreas tumors (n = 13) presented the most discrepant cases, with 69% match between Clinical and MC-Grades, 54% match between Clinical and NQ-Grades and 85% match between MC and NQ-Grades. Two of the 4 discrepant cases put clinical G2 tumors to MC G1. These tumors harboured PIs quite close to the 3% limit, which can be easily overlooked by traditional visual estimation. The other two discrepant cases put the clinical G3 tumors to MC G2 group. Here eyeballing estimated 40% PI, which proved to be below 20% by MC and also with NQ-Grading. This could be explained by the focal and unequal staining of Ki-67 which might have been misleading during clinical grading process. The same cases showed the same G3 to G2 phenomenon between Clinical and NQ-gradings, with the same explanation. As all such cases had lower PIs with the digital methods involving more cells, we believe these discrepancies are showing the vulnerability of estimation/eye-balling. The other 4 G2 to G1 misgradings happened with cases having low proliferation rates around the threshold of 3%. MC and NQ-Grades showed almost perfect agreement (85%), where only 2 cases with PI around 3% (3.12 vs. 2.93 and 3.25 vs. 2.6) were downgraded from MC-Grade 2 to NQ-Grade 1. In contrast to the usual clinical counting/estimation, digital counting is capable to define PIs with more decimal places, an unseen, unexperienced precision level until now. WHO-definition states: G1 means up to 3% PI, while G2 is valid from 3% and decimal precision is not handled by the guideline. Actually, the above mentioned grading discrepancies were caused by not performing rounding numbers, with rounding numbers 100% grading match was achieved in this setting.

With small bowel GEPNENs (n = 22) only one upgrading and one downgrading was found between Clinical and MC or NQ-Gradings, while MC and NQ-Grades showed 100% match. The downgrading put a Clinical PI 3% case to MC/NQ PI 1%–2%, showing again the vulnerability of estimation against digital counting around the low threshold. On the other hand, upgrading happened with a case of Clinical PI 2.5% to digital PI 9%–10%. This might have more clinical relevance and shows that hot-spot localisation can have impact on the final grade. As digital values were higher and based on more cells, we believe that the MC and NQ-Grades must have been more reliable.

Comparing the gradings for all cases (n = 60), the various grading methods showed almost perfect matches (88%, 87% and 95%) as described in the results. MC and NQ-Gradings correlated very strongly, while the clinical grades were sometimes seemingly farer from the digital achieved ones. The reasons for these discrepancies are unfortunately hard to define for each individual case, as clinical grading was done previously and we cannot know how and on which focus it was performed. However, in our opinion, this finding is not unexpected, as clinical grading is typically based on the subjective estimation of pathologists, which inherently depends on their experience and individual interpretative approach. Although guidelines provide recommendations to follow, the exact grading process cannot be reproduced, as pathologists do not document their step-by-step evaluation via video camera or other recording tools. Consequently, it is not possible to exactly replicate the original clinical results. In contrast, MC and NQ-grading provides a more standardized and reproducible approach.

After thorough checking of the discrepant cases we found that majority of these were clinically irrelevant and mainly affected cases around the 3% threshold. In our opinion, these happened due to the previously unexperienced decimal fraction precision of the automated PIs, which opens an unmet field in the precision of Ki-67-assessment. With number roundings, the majority of these discrepancies could have been prevented, showing the problem’s numerical nature without clinical relevance.

Upon comparison of the different grading methods, there was a very strong correlation between the different methods as shown in Table 5 and described in Results.

The strongest correlation of PIs was found between MC and NQ methods and more importantly, the direction of any differences from the original clinical grading was also similar. Furthermore, both MC and NQ-Gradings showed very strong correlations with the clinically/pathologically given proliferation indices, validating each method as a reliable prognosticator. Therefore, we conlcude that all grading methods were equivalent, without clinically relevant differences (Table 5). Considering this equivalency of all methods, the NQ-Grading should be preferable as this method is archivable, its values relies on more cells and can be revisited/recalculated any time, ensuring reproducibility.

The guidelines recommend to perform grading on 500–2000 cells. Accordingly, our study used not less than 2000 (2000–2123, average was 2029) cells for MC-grading, but this approach required a substantial amount of working hours and thus is not easily affordable during routine signout. 2000 cells sound much, even though tumors consist of much more cells and depending on the material (biopsy or surgical) and tumor-pattern (infiltratively or diffusely spreading), a single slide can contain tens or hundreds of thousands cells. Earlier it was shown that tumor heterogeneity, hot-spot localisation/selection and size/shape might have significant influence on Ki-67 PI and thus is a possible source for incorrect PI-definition or interobserver variability. Moreover, the whole tumor global Ki-67 PI delivered more robust prognostic information [8–11, 15, 18].

In lights of these informations, during the automated tumor recognition and Ki-67 counting (NQ grading) we were not limited to the annotations used for the MC-grading and tried to cover bigger tumor areas. The average tumor cell count for NQ-grading was 10,629 (3691–22800) cells. This meant an average 5-fold (with an interval of 2–11 fold) increase to the MC-method, covering much bigger tumorous regions and more closely approximating the global PI.

The strong correlation between MC and NQ Ki-67 values, and thus grades, validated the utilization of the automated method, which, on the other hand, was quick and easy to perform on a regular computer without special assistance, offering a reliable way to help routine signout. Our findings show similar results as other studies on this field, like AIforia-platform in the work of Vesterinen et al, where they found ICC (Intra Class Correlation) of 0.89 between human and digital pathology methods. They found that in 12% of the cases the machine gave slightly lower, while in 42% slightly higher PIs, though their approach used a Convolutional Neural Network model on manual annotations [27]. Goodell et al. compared Mitotic index, single hotspot PI and 10 consecutive field average PI-counting and found that with lower grade tumors/cutoff the single hotspot method delivered lower specificity and prognostic power for metastasis prediction, and the 10 consecutive field was the best. Their study also showed that the number of the involved tumor cells into PI-counting has a significant impact on interobserver variability and prognostication power [40].

In a recent bigger study, Park et al. [32] compared 283 GEPNEN cases with eye-balling and NuclearQuant methods, and also found substantial agreement (*Κ* = 0.765). They used manual hotspot annotation of about 1,000 cells and both of their PI-calculation methods proved to be prognostic according to the Kaplan-Meier curves. They also concluded that digital image analysis (DIA) is a powerful method and parallelly with the adoption of digital pathology into our everyday practice it is capable of easing our workload. DIA offers even greater potential when combined with automated tumor recognition, making it possible to perform whole slide imaging with global Ki-67 score delivering more accurate and robust prognostic power. Earlier studies also showed that increasing the number of involved cells in PI-counting increases the robustness of DIA-based PI [10, 15, 18].

An early work of Reid et al. [7] concluded that eye-balling is rather inaccurate and unreliable, and they recommended replacing it with camera-captured/printed methods. However, they also acknowledged that this method is very laborious, and at that time they regarded automated counting as neither cost-effective nor operator independent. Although, almost a decade has passed from their work which brought tremendous progress in the field of DP and automated cell counting with AI [30]. Our method with PatternQuant and subsequent NuclearQuant automated Ki-67 PI-calculation offers a very quick and reliable method suitable for incorporation into routine application.

According to WHO, the differentiation and size of the GEPNEN cells could be also used as information for grading, as Grade 3 tumors can be put into Well Differentiated Neuroendocrine Tumors (WD-NET) or Poorly differentiated Neuroendocrine Carcinomas (PD-NEC) depending on mitotic figures, PIs, but also on cell size and cellular features discriminating between small and large cell neuroendocrine carcinomas. Still, we are not aware of any study investigating the cytomorphological parameters in GEPNENs.

We measured the averages and standard deviations of tumor cell nuclear area, perimeter and shape factor of all the involved 60 cases while performing the NQ-counting and found that these significantly differed between G1/G3 and G2/G3 classes with either grading methods (Clinical, MC or NQ). There were also more significant differences between G1 and G2 classes, especially in the standard deviation of the values. Table 7; Figure 4 shows that G3 tumor cells have significantly larger nuclei and nuclear perimeter than G1 and G2 tumor cells, independently from the grading method (Clinical, MC and NQ). Furthermore, the significantly smaller shape factor of the G3 tumors described a more variable shape of the polymorphic high grade tumor cells. These findings are in line with the WHO guidelines, though their utilization would need more research, as in our cohort, the size and shape data were not enough to separate the tumors into different grades. Perhaps a more sophisticated or combined analysis, involving more parameters of the tumor cells might deliver better classification opportunities/perspectives.

There are certain limitations of our study. First is the relative low number of the 60 involved cases, but this was the maximum accessible surgical material in our archive. We excluded biopsies because those might not contain 2000 cells. The second limitation is the lack of follow up data, which could be the final answer for demonstrating the prognostic power of the various grading methods. It is our future plan to correlate the digitally derived PIs with patients’ follow up data and show their robustness on Kaplan-Meier curves and with Cox regression analysis. The additional data that tumors of various grades differ from each other in their size and shape, might be also utilized later, but our study cohort did not allow the separate subanalyis of WD-NETs and PD-NECs or to classify tumors upon their nuclear size or parameters. With follow up data the cytomorphological parameters can be also tested towards their prognostic power or relevance.

## Conclusion

Our smaller, retrospective, single-centered study showed that the automatic tumor-recognition and Ki-67 counting method offered by a simple machine learning algorithm, PatternQuant and NuclearQuant (3Dhistech, Hungary, Budapest), delivers equivalent grading of GEPNENs with the traditional methods. Our results validated that automated PI assessment is achievable with digital pathology and has the potential to replace the rather demanding manual counting methods, thus easing routine pathology signout process with increased precision by involving more cells to better approximate a more reliable global PI and potentially enable the use of additional cytomorphological parameters.

Compliance with Ethical Standards: All clinical data, slides and specimens were handled in a coded manner for anonymization. In accordance with the Hungarian Law, the ethical guidelines of the country and the Helsinki Declaration, no formal consent was required from our patients to this retrospective, non-interventional study not affecting patients’ therapy. All data and samples were dealt with the approval of the Hungarian Scientific Council National Ethics Committee for Scientific Research (no. 216/2020). This research is the authors’ own work, which has not yet been published elsewhere. This is paper is not being currently considered for publication elsewhere.

## Data Availability

The raw data supporting the conclusions of this article will be made available by the authors, without undue reservation.
